# Effects of an 8-Week Mindfulness Course on Affective Polarization

**DOI:** 10.1007/s12671-021-01808-0

**Published:** 2022-01-07

**Authors:** Otto Simonsson, Olivier Bazin, Stephen D. Fisher, Simon B. Goldberg

**Affiliations:** 1 Center for Healthy Minds, University of Wisconsin, Madison, Madison, WI USA; 2 Department of Sociology, University of Oxford, Oxford, UK; 3 Department of Counseling Psychology, University of Wisconsin - Madison, Madison, WI USA; 4 British Association of Mindfulness-based Approaches (BAMBA) Listed Teacher, Oxford, UK

**Keywords:** Polarization, Intergroup, Political, Brexit, Mindfulness, Meditation

## Abstract

**Objectives:**

The European Union Brexit referendum has split the British electorate into two camps, with high levels of affective polarization between those who affiliate with the Remain side (Remainers) and the Leave side (Leavers) of the debate. Previous research has shown that a brief meditation intervention can reduce affective polarization, but no study has thus far investigated the effects of an 8-week mindfulness program on affective polarization. This is what will be examined in this study.

**Methods:**

The present study used a randomized waitlist control design (*n* = 177) with a 1-month post-intervention follow-up to investigate whether an 8-week mindfulness program delivered online would have an effect on affective polarization among Remainers and Leavers.

**Results:**

Results showed significantly greater reductions in affective polarization over time for participants in the mindfulness condition relative to participants in the waitlist control condition (time X group *B* =  − 0.087, *p* = .024).

**Conclusions:**

Taken together, the findings highlight the potential of mindfulness training as a means to reduce intergroup biases in political contexts.

**Trial Registration:**

Preregistered on the Open Science Framework at https://osf.io/px8m2.

In a nationwide referendum on June 23, 2016, the British electorate was asked whether the United Kingdom (UK) should remain or leave the European Union (EU), with the majority of votes (51.9%) cast in favor of leaving the EU (Brexit). Although the question of EU membership had not been particularly salient before the referendum, the opinion-based identities associated with Brexit (i.e., Remainers and Leavers) have been shown to be stronger and more widespread than traditional party identities, even years after the referendum (Hobolt et al., 2021).

The evidence to date suggests that Remainers and Leavers, on an aggregate level, have high levels of affective polarization (Hobolt et al., 2021) — defined as the difference in feelings and perceptions towards the political ingroup and the political outgroup (Iyengar et al., 2019). Given that a degree of mutual respect and willingness to engage in political discussion is essential for the democratic process, it is not surprising that high levels of affective polarization have been associated with political consequences that could be harmful to the functioning of democracy (Abramowitz & Webster, 2016; Druckman et al., 2021; Hetherington & Rudolph, 2015). Hence, it is important to investigate scalable and accessible interventions to reduce affective polarization.

Previous research has investigated a range of psychological interventions to reduce affective polarization (Garrett et al., 2014; Huddy & Yair, 2021; Iyengar et al., 2019; Levendusky, 2018; Warner et al., 2020; Wojcieszak & Garrett, 2018). For example, one intervention that has shown considerable promise is imagined intergroup contact (Crisp & Turner, 2009; see also Pettigrew, 1998 for intergroup contact theory), which involves imagining positive social interactions with outgroup members. There is a growing body of research showing that imagined intergroup contact can improve intergroup attitudes more generally (Miles & Crisp, 2014), but recent research has also shown that it can decrease affective polarization between Democrats and Republicans (Warner & Villamil, 2017; Wojcieszak & Warner, 2020).

Yet, the explicit emphasis on outgroup members in imagined intergroup contact could be a barrier in real-world situations when affective polarization is high. For instance, people with high levels of affective polarization may be reluctant to voluntarily engage in an exercise that explicitly asks them to imagine the political outgroup, especially if the political outgroup represents a symbolic or physical threat to the political ingroup. Other psychological interventions without explicit emphasis on bias reduction may therefore be useful to mitigate affective polarization.

The practice of mindfulness meditation has become increasingly popular in recent decades, and a range of secular mindfulness programs have been developed for different populations. Such programs have been introduced in schools, businesses, prisons (Creswell, 2017), and even the UK Parliament (Bristow, 2019), where politicians from across the political spectrum have attended an 8-week mindfulness program adapted from Mindfulness-Based Cognitive Therapy (MBCT; Segal et al., 2018): the Finding Peace in a Frantic World curriculum (Williams & Penman, 2011). While the Finding Peace in a Frantic World curriculum has been shown to have mental health benefits (Galante et al., 2018), many of the elected officials have reported other benefits that are directly related to politics, including greater empathy with constituents, better self-regulation in adversarial situations, and more skillful engagement with differing opinions and views (Bristow, 2019). It suggests that mindfulness practice may be helpful in the political arena (see Bristow, 2019; Ferguson, 2016; Kabat-Zinn, 2005; Klein, 2020; McLeod, 2006; Moore, 2016; Ryan, 2012 for discussions on mindfulness in politics).

The anecdotal reports from politicians have recently been followed by experimental research on how affective polarization may be affected by befriending meditation, which is one of the practices taught in the Finding Peace in a Frantic World curriculum (Williams & Penman, 2011). Results from previous research have shown that 10 min of befriending meditation reduced affective polarization in American adults who affiliated with either the Democratic Party or the Republican Party (Simonsson et al., 2021). While promising, effects in these studies were modest in magnitude (*d*s ≤ 0.31). The meditation inductions were brief and therefore left open the possibility that effects were state-related and short-lived. While other research suggests that mindfulness training may reduce intergroup bias more generally (Oyler et al., 2021), the effects of sustained mindfulness training on affective polarization measured outside the context of a practice session and at longer-term follow-up so far remain unknown.

Using a randomized waitlist control design, we investigated whether the Finding Peace in a Frantic World curriculum (delivered online) had an effect on affective polarization among Remainers and Leavers. We hypothesized that participants randomly assigned to the mindfulness condition would show a decrease in affective polarization relative to participants randomly assigned to the waitlist control condition.

## Methods

### Participants

Students aged 18 years or older at the University of Oxford were eligible for the study, which was advertised through emails and social media. The mindfulness course was offered for free to students at participating colleges. The interested students were given more details about the study and were invited to give their consent to participate through Qualtrics (https://www.qualtrics.com/), the platform used to collect the data for the study.

Sample size was determined using a power analysis (G*Power Version 3.1.9.2; Faul et al., 2007). We assumed a medium-sized between-group effect (i.e., independent *t*-test on change scores) and found that a sample size of 128 participants would achieve 80% power to detect an effect size *d* = 0.50 with α = 0.05. Assuming 20% attrition, we aimed to recruit at least 160 participants.

**Fig. 1:**
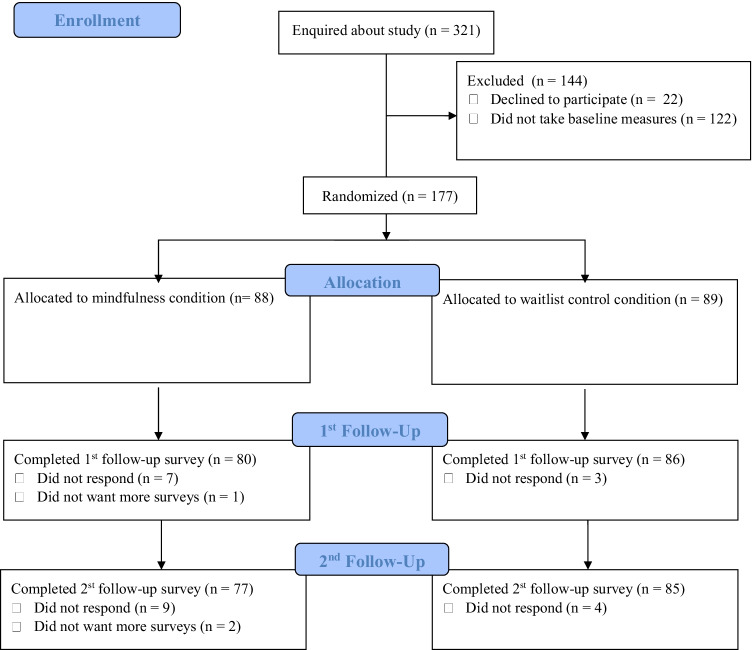
Participant flow diagram

Sample demographics are reported in Table 1. We randomized 177 participants (95 British citizens, 38 EU citizens, 44 other; 114 females, 56 males, 4 prefer not to say, 3 other write-in section: 2 non-binary, 1 genderfluid; aged 18–57; M = 23.53, SD = 6.16). Of these, 166 (94%) completed post-test measures and 162 (92%) 1-month follow-up (see Fig. 1 for a study flow diagram).

**Table 1:** Sample demographic characteristics

Variables	Mean	SD	%	n	Min	Max	Skew	Kurtosis
Age	23.53	6.16			18	57	2.86	13.96
Female			64.41	114	0	1	− 0.60	1.36
British citizen			53.67	95	0	1	− 0.15	1.02
White			68.93	122	0	1	− 0.82	1.67
Undergraduate			55.93	99	0	1	− 0.24	1.06
English first language			69.49	123	0	1	− 0.85	1.72
Past meditation experience			63.84	113	0	1	− 0.58	1.33
Past mindfulness course			5.65	10	0	1	3.84	15.76
Study completion likelihood	6.35	1.03			1	7	− 3.22	15.95
Remainer identity			93.79	166	0	1	− 3.63	14.16
Strength of Brexit identity	3.33	0.59			2	4.8	0.04	2.55

The table describes sample characteristics for the full sample (*n* = 177)

### Procedures

Eligible participants completed baseline assessments (T1) prior to randomization in January 2021. Randomization was conducted using the excel randomization formula with participants randomly assigned (1:1) to begin the Finding Peace in a Frantic World curriculum immediately (i.e., intervention arm) or following the end of the study (i.e., waitlist control). Post-test assessment (T2) occurred 8 weeks following baseline assessment (i.e., at the end of the Finding Peace in a Frantic World curriculum). A follow-up assessment (T3) was conducted 1 month later.

The intervention was an 8-week secular, instructor-led, group-based skills training program, adapted from the book *Mindfulness: a Practical Guide to Finding Peace in a Frantic World* (Williams & Penman, 2011), which has also been delivered to politicians in the UK Parliament. The course was delivered via the Zoom platform (https://zoom.us/) and consisted of eight weekly classes, each of which lasted for 90 min (sessions 1 and 2 were 105 min long, to allow the participants to become familiar with the online format). The classes involved mindfulness meditation exercises, periods of reflection and inquiry, and interactive exercises drawn from cognitive behavioral therapy. The participants were also encouraged to set aside 20–30 min per day for home practice (see Table 2 for an overview of the program).

**Table 2:** Overview of Finding Peace in a Frantic World curriculum

Week	Theme	In-session practices
1	Waking up to the autopilot	Mindful eating exercise, mindfulness of body and breath
2	Keeping the body in mind	Body scan, mindful speaking and listening, appreciation exercise
3	Mindfulness in daily life	3-step breathing space, mindful movement, mindfulness of breath and body
4	Relating differently to thoughts and worries	Scenario-based thoughts and feelings exercise, mindfulness of sounds and thoughts
5	Turning towards difficulties	Reflection on the cost of reactivity, working with difficulty meditation, extended breathing space to include the difficult
6	Practising kindness	Working with difficulty, scenario-based exercise on recognizing self-critical habits and patterns, befriending meditation
7	Developing balance in our lives	Nourishing and depleting activities, rebalancing exercises, breathing space + action step, behavioral activation
8	Intentions for practice	Reflective course review, mindful speaking and listening, writing “letter to self” and personal practice plan

Sessions follow Chapters 5–12 in sequence in the course book (Williams & Penman, 2011)

There were four courses delivered in parallel, with 15 to 29 participants in each group. All courses were taught by the same mindfulness teacher. The mindfulness teacher met Good Practice Guidelines for teachers of Mindfulness-Based Interventions (BAMBA, 2021) and was also blind to the research focus and hypothesis. If participants were absent from a class, the mindfulness teacher would contact them to check if they needed support and to invite them to attend one of the remaining parallel sessions. Participants were also offered the opportunity to speak with the instructor in between sessions if they were experiencing difficulties.

### Measures

#### Demographics

Students who agreed to participate in the study were asked to provide demographic information (gender, age, citizenship, ethnicity, first language, degree program, previous experience with meditation, and previous experience attending an 8-week mindfulness course).

#### Brexit Identity

Brexit identity was assessed using the following question: “On 23 June 2016, the United Kingdom held a referendum to ask the electorate whether the country should remain a member of, or leave, the European Union (EU). In the EU referendum debate, do you think of yourself as closer to either the Remain or Leave side?” Participants were subsequently presented with five items designed to measure their strength of identity with whichever side they were closer to (“When people criticize the […] side, it feels like a personal insult”; “When I speak about the […] side, I usually say “we” instead of “they””; “When people criticize the […] side, it feels like a personal insult”; “I have a lot in common with other supporters of the […] side”; “When I meet someone who supports the […] side, I feel connected with this person”; “When people praise the […] side, it makes me feel good”; Hobolt et al., 2021). The responses were rated on a 1- (Strongly disagree) to 5-point (Strongly agree) Likert scale. A total score was computed by summing across the five items (*α* = 0.73), with a higher score indicating stronger Brexit identity.

#### Dispositional Mindfulness

As a manipulation check, participants completed the 15-item Five-Facet Mindfulness Questionnaire (FFMQ-15; Gu et al., 2016), which is a short-form version of the 39-item FFMQ (Baer et al., 2006). Both the 15- and 39-item FFMQ are designed to assess dispositional mindfulness. They have shown high internal consistency reliability, convergent validity (e.g., with depression and rumination), and change in response to meditation training. The FFMQ consists of five subscales: observing (e.g., “When I take a shower or a bath, I stay alert to the sensations of water on my body”), describe (e.g., “I’m good at finding words to describe my feelings”), acting with awareness (e.g., reverse-scored: “I don’t pay attention to what I’m doing because I’m daydreaming, worrying, or otherwise distracted”), nonjudging (e.g., reverse-scored: “I believe some of my thoughts are abnormal or bad and I shouldn’t think that way”), and nonreactivity (e.g., “When I have distressing thoughts or images I just notice them and let them go”). As recommended (Gu et al., 2016), a total score was computed by summing across all items with the exception of the observing subscale (*α* = 0.56). The responses were rated on a 1- (Never or very rarely true) to 5-point (Very often or always true) Likert scale, with higher scores indicating higher dispositional mindfulness.

#### Affective Polarization

The feeling thermometer (Iyengar et al., 2019; Lelkes & Westwood, 2017) is a widely used measure of affective polarization. Participants were asked to indicate, on a scale ranging from cold (0) to warm (100), their feelings toward their own side in the EU Referendum debate and the rival side in the EU Referendum debate. The difference between these two ratings (i.e., thermometer rating for own side minus rating for rival side) was computed, with higher scores indicating higher affective polarization.

Trait ratings (Iyengar et al., 2012, 2019) have also been widely used to assess affective polarization. Participants indicated how well they thought different traits (intelligent, honest, open-minded, generous; reverse-scored: hypocritical, selfish, mean) applied to their own side in the EU Referendum debate and the rival side in the EU Referendum debate. Patriotism has been included as a trait in previous studies with US samples, but as patriotism is not necessarily a virtue in Europe or the UK, a total score was computed by summing across all items with the exception of the patriotism item (*α* = 0.59). The responses were rated on a 1- (Not at all well) to 5-point (Extremely well) Likert scale. A total score was computed by summing across items for participants’ own side and the rival side. As before, the difference between these two scores was computed, with higher scores indicating higher affective polarization.

Affective polarization scores for each measure at each time point were computed by subtracting ratings for the rival side in the EU Referendum debate from ratings for their own side. Thus, higher scores indicated greater ingroup preference and higher affective polarization. Consistent with our preregistration, a composite of scores on the feeling thermometer and trait ratings served as our measure of affective polarization and dependent variable. These measures were highly correlated at baseline (*r* = 0.62, *p* < 0.001). In order to create a single measure of affective polarization, scores were first z-transformed using the baseline mean and standard deviation for each variable, respectively, before being averaged into a single item.

### Data Analyses

We used multilevel modeling with observations nested within participants over time as our primary analytic strategy. We used the ‘lmer’ function in the ‘lme4’ package (Bates et al., 2015) in R (R Core Team, 2021). Restricted maximum likelihood estimation was used which is robust to data that are missing at random (Graham, 2009). The impact of randomization on affective polarization (dependent variable) and dispositional mindfulness (manipulation check) was assessed through examining the interaction between group assignment and time. Analyses were conducted using the intention-to-treat sample (i.e., with all randomized participants included, regardless of their completion of post-test or follow-up assessments). Participants who reported neither British nor EU citizenship were excluded from the primary analysis on affective polarization based on the assumption that Brexit identity may be less salient for them.

We conducted three preregistered sensitivity analyses. These included running our primary analysis with all participants included, with outliers excluded (i.e., affective polarization scores three or more standard deviations from the mean), and restricted to completers.

## Results

Independent samples *t*-tests and chi-square tests revealed that there were no significant differences across conditions on any of the variables at baseline (see Table 3). Groups did not differ in their likelihood of completing post-test or follow-up assessments (*ps* > .050). Descriptive statistics for study variables are reported in Tables 4 and 5.

**Table 3:** Between-group differences at baseline

Variables	*t*	*χ*^2^	*p*
Age	0.81		.419
Gender		4.61	.202
Citizenship		1.08	.584
Ethnicity		3.37	.498
Degree program		1.24	.744
English first language		0.36	.546
Past meditation experience		0.07	.798
Past mindfulness course		0.00	.985
Study completion likelihood		7.34	.290
Brexit identity		2.36	.124
Strength of Brexit identity	1.49		.138
Observing	− 0.32		.753
Describe	− 0.84		.403
Acting with awareness	− 0.07		.945
Nonjudging	− 0.22		.822
Nonreactivity	− 0.04		.971
FFMQ	− 0.46		.694
Feeling thermometer	− 1.16		.247
Trait ratings	− 1.10		.274
Affective polarization	− 1.26		.211

The table describes baseline differences for the full sample (*n* = 177). Independent samples *t*-tests are used to assess baseline differences for continuous variables and chi-square tests are used to assess baseline differences for categorical variables. The affective polarization variable is made up of the feeling thermometer and trait ratings (see the “Methods” section for more information)

**Table 4:** Outcome measures and manipulation check at pre, post, and follow-up

Variables	Mean	SD	Min	Max	Skew	Kurtosis
T1: Mindfulness group (*n* = 88)						
Observing	3.21	0.75	1.67	4.67	− 0.18	− 0.59
Describe	3.40	0.83	1.33	5.00	− 0.40	− 0.16
Acting with awareness	2.68	0.67	1.00	4.00	− 0.10	− 0.14
Nonjudging	3.10	0.88	1.00	5.00	− 0.01	0.70
Nonreactivity	2.69	0.75	1.00	4.67	− 0.10	0.17
FFMQ	2.97	0.53	1.33	4.08	− 0.42	0.03
Feeling thermometer	42.74	31.99	− 31	100	− 0.07	− 0.87
Trait ratings	0.95	0.90	− 1.71	3.43	0.08	0.16
Affective polarization	0.09	0.86	− 2.05	2.18	0.03	− 0.52
T1: Control group (*n* = 89)						
Observing	3.17	0.78	1.00	4.67	− 0.59	0.25
Describe	3.30	0.79	1.67	5.00	0.12	− 0.69
Acting with awareness	2.67	0.79	1.00	5.00	0.00	− 0.23
Nonjudging	3.07	0.96	1.00	5.00	− 0.04	− 0.69
Nonreactivity	2.69	0.73	1.33	5.00	0.57	0.16
FFMQ	2.93	0.52	1.58	4.00	− 0.24	− 0.06
Feeling thermometer	37.07	32.98	− 100	100	− 1.08	2.51
Trait ratings	0.79	1.03	− 2.29	3.57	− 0.08	0.87
Affective polarization	− 0.08	0.94	− 3.33	2.32	− 0.58	1.77
T2: Mindfulness group (*n* = 80)						
Observing	3.63	0.64	2.00	5.00	0.08	− 0.12
Describe	3.55	0.75	1.67	5.00	− 0.01	− 0.31
Acting with awareness	3.00	0.63	1.33	4.67	0.08	0.74
Nonjudging	3.56	0.82	2.00	5.00	− 0.09	− 0.76
Nonreactivity	3.25	0.66	1.67	5.00	0.32	0.21
FFMQ	3.34	0.51	2.25	4.92	0.22	0.53
Feeling thermometer	31.06	34.45	− 84	100	− 0.40	0.13
Trait ratings	0.95	0.86	− 2.14	2.57	− 1.24	2.68
Affective polarization	− 0.09	0.84	− 2.16	1.8	− 0.49	− 0.09
T2: Control group (*n* = 86)						
Observing	3.30	0.83	1.00	5.00	− 0.32	− 0.05
Describe	3.33	0.74	1.33	5.00	− 0.02	− 0.02
Acting with awareness	2.80	0.78	1.00	4.67	− 0.12	− 0.09
Nonjudging	3.11	0.97	1.00	5.00	− 0.42	− 0.52
Nonreactivity	2.74	0.83	1.00	4.67	0.18	− 0.33
FFMQ	3.00	0.57	1.58	4.42	− 0.01	− 0.11
Feeling thermometer	38.87	35.25	− 100	100	− 0.89	2.10
Trait ratings	1.03	1.00	− 2.71	2.57	− 1.56	2.56
Affective polarization	0.07	0.96	− 3.55	1.29	− 1.55	3.10
T3: Mindfulness group (*n* = 77)						
Observing	3.54	0.63	1.67	4.67	− 0.28	0.12
Describe	3.55	0.69	1.67	5.00	− 0.22	− 0.37
Acting with awareness	2.88	0.62	1.00	4.00	− 0.13	0.23
Nonjudging	3.52	0.95	1.33	5.00	− 0.28	− 0.64
Nonreactivity	3.09	0.63	1.33	4.67	− 0.21	0.60
FFMQ	3.26	0.51	1.75	4.33	− 0.32	0.40
Feeling thermometer	32.22	30.73	− 48	100	0.02	− 0.50
Trait ratings	0.95	0.77	− 1.29	2.86	− 0.45	0.22
Affective polarization	− 0.08	0.75	− 1.92	1.95	0.01	− 0.21
T3: Control group (*n* = 85)						
Observing	3.36	0.86	1.00	5.00	− 0.26	0.04
Describe	3.33	0.80	1.33	5.00	− 0.15	− 0.61
Acting with awareness	2.69	0.84	1.00	5.00	− 0.08	− 0.22
Nonjudging	3.23	0.95	1.00	5.00	− 0.48	− 0.32
Nonreactivity	2.83	0.84	1.00	4.67	0.07	− 0.96
FFMQ	3.02	0.60	1.25	4.08	− 0.44	− 0.04
Feeling thermometer	32.48	35.06	− 85	100	− 0.50	0.83
Trait ratings	0.94	0.85	− 1.57	2.57	− 0.91	0.77
Affective polarization	− 0.08	0.87	− 2.85	1.8	− 0.69	0.72

The affective polarization variable is made up of the feeling thermometer and trait ratings (see the “Methods” section for more information)

**Table 5:** Within- and between-group changes in outcome measures and manipulation check

		Within-group *d*s		Between-group *d*s	
Variable	Group	T1-to-T2	T1-to-T3	T1-to-T2	T1-to-T3
Observing	Mindfulness	0.60	0.48	0.44	0.26
	Control	0.16	0.22		
Describe	Mindfulness	0.19	0.19	0.15	0.14
	Control	0.04	0.05		
Acting with Awareness	Mindfulness	0.48	0.31	0.32	0.29
	Control	0.16	0.02		
Nonjudging	Mindfulness	0.55	0.47	0.50	0.30
	Control	0.05	0.17		
Nonreactivity	Mindfulness	0.80	0.57	0.72	0.39
	Control	0.08	0.18		
FFMQ	Mindfulness	0.72	0.56	0.60	0.40
	Control	0.12	0.16		
Feeling thermometer	Mindfulness	− 0.35	− 0.33	− 0.40	− 0.20
	Control	0.05	− 0.13		
Trait ratings	Mindfulness	0.00	0.00	− 0.24	− 0.16
	Control	0.24	0.16		
Affective polarization	Mindfulness	− 0.21	− 0.20	− 0.37	− 0.19
	Control	0.16	− 0.01		

Effect sizes are measured as Cohen’s *d*; the affective polarization variable is made up of the feeling thermometer and trait ratings (see the “Methods” section for more information). Scores on feeling thermometer, trait ratings, and affective polarization composite calculated as the differences between rating for political ingroup minus political outgroup (i.e., higher scores indicate higher affective polarization). Positive within-group *d*s indicate increases in construct and negative *d*s indicate decreases in construct. Positive between-group *d*s indicate relatively larger increases in construct in the mindfulness condition relative to control condition. Negative between-group *d*s indicate relatively larger decreases in construct in the mindfulness condition relative to control condition

We first evaluated the effect of our manipulation by examining between-group differences in changes in dispositional mindfulness over time. A significant time X group interaction was detected for changes in dispositional mindfulness, with the mindfulness arm showing significantly greater increases over time relative to the waitlist condition (time *B* = 0.034, *p* = .075; group *B* = 0.032, *p* = .742; time X group *B* = 0.084, *p* = .002). A significant time X group interaction was also detected for changes in the nonjudging and nonreactivity subscales, with the mindfulness arm showing significantly greater increases over time relative to the waitlist condition (time *B* = 0.050 and 0.055, *p* = .153 and .066, group *B* = 0.025 and 0.035, *p* = .879 and .797, time X group *B* = 0.10 and 0.12, *p* = .034 and .005, respectively). However, no significant time X group interactions were detected for changes in the other FFMQ subscales (*ps* > .050).

Between-group differences in affective polarization were examined next, initially restricting to British and EU citizens (*n* = 133). A significant time X group interaction was detected for changes in affective polarization, with the mindfulness arm showing significantly greater reductions over time relative to the waitlist condition (time *B* = 0.003, *p* = .905; group *B* = 0.090, *p* = .527; time X group *B* =  − 0.087, *p* = .024). The time X group interaction remained significant and the coefficient essentially unchanged when including the full sample (i.e., not restricted to British or EU citizens; time X group *B* =  − 0.075, *p* = .030), when excluding outliers (*n* = 2 outliers removed at each time point; time X group *B* =  − 0.085, *p* = .029), and when restricted to those completing both post-test and follow-up assessments (*n* = 120; time X group *B* =  − 0.081, *p* = .040).

## Discussion

Using a randomized waitlist control design, we investigated whether an 8-week mindfulness program had an effect on affective polarization among Remainers and Leavers. The mindfulness arm showed a significant decrease in the trajectory of change over time relative to the waitlist control arm, which corresponds with previous findings on the relationships between mindfulness training and other types of intergroup bias (Oyler et al., 2021). It suggests that sustained mindfulness training may be an effective intervention to reduce affective polarization, at least modestly. Importantly, in contrast to studies testing meditation inductions on affective polarization (e.g., Simonsson et al., 2021), these effects were observed outside the context of meditation practice. This supports the possibility that meditation training may lead to sustained and generalized changes in attitudes toward outgroup members. While the effects were small, they are notable given the importance of affective polarization and the fact that the intervention did not actually target affective polarization explicitly (i.e., were not directly manipulated; Prentice & Miller, 1992).

This study investigated how affective polarization may be affected by the Finding Peace in a Frantic World curriculum, which has been taught to British MPs and Lords in the UK Parliament (Bristow, 2019). The link to a real-world setting makes the study particularly relevant and suggests that mindfulness training may have benefits for elected officials beyond promotion of mental health. Indeed, anecdotal reports suggest that mindfulness training has helped politicians to cope with challenges that are fairly unique to the political process (Bristow, 2019). Future studies could use qualitative methods to better understand how mindfulness training may have helped politicians to perform their daily duties as elected officials. Future quantitative work could also clarify the mechanisms by which meditation training may reduce affective polarization. It would be valuable to replicate the current study using a highly scalable self-guided meditation intervention (e.g., delivered via smartphone app; Gál et al., 2021), which could represent a feasible pathway to effecting changes in social discourse.

### Limitations and Future Research

There are several limitations that are important to consider. First, participants self-selected into the study and most participants identified as Remainers, which limits the generalizability of the findings. Second, strength of Brexit identity was assessed, but participants were not asked whether they voted in the Brexit referendum or not. It is possible that effects may have differed between voters and non-voters. Third, home practice was not assessed and intervention effects may therefore have varied across participants depending on the degree of home practice, as has been observed for changes in other outcomes (Parsons et al., 2017). It might also have varied had the intervention been delivered outside the context of live group sessions. Fourth, the coronavirus disease (COVID-19) pandemic was ongoing during the study and the UK officially left the EU on January 31, 2021, between the study’s T1 and T2 assessments. These historical events may have impacted the study results (e.g., increased affective polarization in the control condition from T1 to T2). Fifth, the use of a waitlist control condition limited the risk of a temporal confound, but it did not control for the influence of non-specific factors (e.g., instructor attention, expectancy). Sixth, the dependent variable was a self-report measure and therefore susceptible to a range of biases such as social desirability bias. Future studies on the effects of long-term meditation training on affective polarization should include active control groups and behavioral measures. It would be particularly valuable to clarify necessary dosage and delivery format as well as examine whether meditation training influences actual behaviors linked to affective polarization (e.g., interactions on social media; Yarchi et al., 2021).

## Data Availability

The data and R script are available at the Open Science Framework: https://osf.io/rxf87/files.
